# Chili Pepper *AN2* (*CaAN2*): A Visible Selection Marker for Nondestructive Monitoring of Transgenic Plants

**DOI:** 10.3390/plants11060820

**Published:** 2022-03-19

**Authors:** Sun-Hyung Lim, Da-Hye Kim, Myeong-Cheoul Cho, Jong-Yeol Lee

**Affiliations:** 1 Division of Horticultural Biotechnology, School of Biotechnology, Hankyong National University, Anseong 17579, Korea; kimdh143@naver.com; 2 Research Institute of International Technology and Information, Hankyong National University, Anseong 17579, Korea; 3 National Institute of Horticultural and Herbal Science, Rural Development Administration, Wanju 55365, Korea; chomc@korea.kr; 4 National Institute of Agricultural Sciences, Rural Development Administration, Jeonju 54874, Korea

**Keywords:** alternative selection method, anthocyanin, *CaAN2*, *Capsicum annuum*, transformation, visible marker

## Abstract

Selecting transformed plants is generally time consuming and laborious. To develop a method for transgenic plant selection without the need for antibiotics or herbicides, we evaluated the suitability of the R2R3 MYB transcription factor gene *CaAN2* from purple chili pepper (*Capsicum annuum*) for use as a visible selection marker. *CaAN2* positively regulates anthocyanin biosynthesis. Transient expression assays in tobacco (*Nicotiana tabacum*) leaves revealed that *CaAN2* actively induced sufficient pigment accumulation for easy detection without the need for a basic helix-loop-helix (bHLH) protein as a cofactor; similar results were obtained for tobacco leaves transiently co-expressing the anthocyanin biosynthesis regulators bHLH B-Peru from maize and R2R3 MYB mPAP1D from Arabidopsis. Tobacco plants harboring *CaAN2* were readily selected based on their red color at the shoot regeneration stage due to anthocyanin accumulation without the need to impose selective pressure from herbicides. Transgenic tobacco plants harboring *CaAN2* showed strong pigment accumulation throughout the plant body. The ectopic expression of *CaAN2* dramatically promoted the transcription of anthocyanin biosynthetic genes as well as regulators of this process. The red coloration of tobacco plants harboring *CaAN2* was stably transferred to the next generation. Therefore, anthocyanin accumulation due to *CaAN2* expression is a useful visible trait for stable transformation, representing an excellent alternative selection system for transgenic plants.

## 1. Introduction

Pepper (*Capsicum annuum*) is an economically important vegetable that provides antioxidant compounds (with anti-inflammatory and antimicrobial effects) for the human diet. Engineering the pepper genome to produce novel and useful agronomic traits requires development of stable transformation methods and accurate selectable markers. Various selectable markers have been used for crop transformation, with their transformation efficiencies being strongly affected by the type of marker chosen [1,2]. The use of antibiotic and herbicide resistance genes as positive selection markers has prompted biosafety concerns about human health and the environment [3,4]. To address these concerns, β-glucuronidase and fluorescent proteins are generally used for the identification of transformed cells. However, these systems have several limitations, such as the need for destructive GUS staining methods and for expensive equipment to detect fluorescent signals [5,6]. Additionally, the process of genetic transformation and regeneration is time consuming and labor intensive, in terms of selection and characterization of transformed cells and occasionally can result in chimerism (a single plant tissue containing transformed and non-transformed sections), thus requiring additional experiments for gene transfer to subsequent generations.

Anthocyanins, a large class of secondary metabolites, are widely distributed in various plant tissues, including flowers, stems, leaves and fruits, with colors ranging from red to blue [7]. Therefore, anthocyanins could potentially be used as a selectable marker for the visual identification of transformed cells during in vitro culture. The anthocyanin biosynthetic pathway involves a multienzyme complex and is controlled by key transcription factors, including R2R3-MYB, basic helix-loop-helix (bHLH) and WD40 proteins, as well as MBW complexes [8,9]. The R2R3-MYB transcription factors, belonging to subgroup 5 (SG5) and SG6, activate anthocyanin biosynthesis. The ectopic expression of the R2R3-MYB transcription factor genes in apple, barrelclover, radish and strawberry leads to red or purple coloration in various tissues, including calli, root tips and leaves, by upregulating the expression of anthocyanin biosynthetic genes [10,11,12].

Most pepper plants have green stems and leaves, white flowers and fruits that turn from green to red at maturity; others have purple stems, leaves, flowers and fruits at the immature stage and red fruits at the ripe stage. *CaAN2* (*ANTHOCYANIN2*), an ortholog of petunia *PhAN2*, was isolated from purple pepper and shown to be responsible for the skin color of purple pepper fruits [13]. In purple-fruited pepper, variation in the *CaAN2* promoter region can enhance the expression level of this gene in various tissues, resulting in the accumulation of anthocyanin pigments [14].

In this study, to investigate the potential use of *CaAN2* as a visible selectable marker, a transient assay and stable transformation of tobacco (*Nicotiana tabacum*) plants was performed. The ectopic expression of *CaAN2* induced anthocyanin accumulation in tobacco. In addition, tobacco plants that were stably transformed with *CaAN2* showed strong pigment accumulation, which was steadily transferred to the next generation. These results indicate that *CaAN2* could be utilized as an alternative visible selectable marker to facilitate transgenic plant identification.

## 2. Results

### 2.1. Anthocyanin Accumulation Determines the Green and Purple Coloration of Chili Pepper

**Figure 1:**
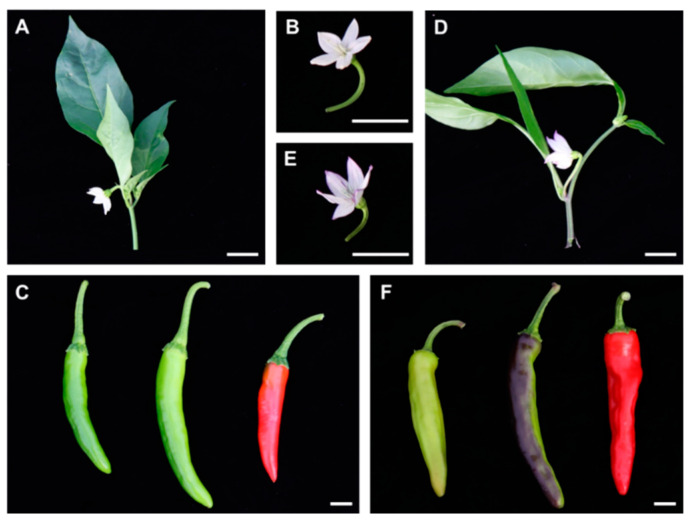
Comparison of the phenotypes of green (cv. AG188) and purple chili pepper (cv. 20GP15-2). (**A**) Green pepper plant; (**B**) green pepper flower; (**C**) green pepper fruits at different stages of development: mature green (left), breaker (middle) and red-ripe (right). (**D**) Purple pepper plant; (**E**) purple pepper flower; and (**F**) purple pepper fruits at different stages of development. Bars = 1 cm.

To investigate the mechanisms controlling anthocyanin biosynthesis in chili pepper, two pepper cultivars with different pigmentation patterns in fruits were analyzed. The green cultivar (G) has green leaves, green stems and white flowers, and its fruits are green at the mature green fruit stage 1 (FS1) and gradually become red at the red ripe stage (FS3). The purple cultivar (P) has green leaves, purple stems and purple flowers and its fruits are green at FS1, turn purple at the breaker stage (FS2) and are red at FS3 (Figure 1).

**Figure 2:**
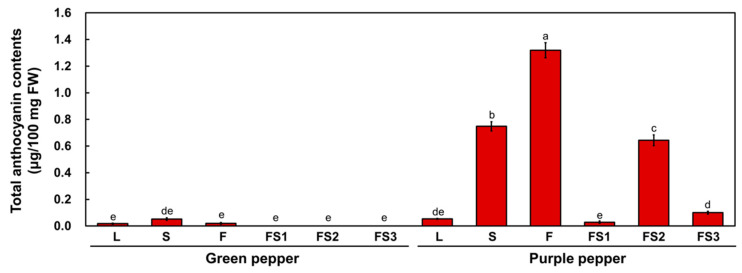
Anthocyanin contents of various green and purple pepper tissues. L, leaves; S, stems; F, flowers; FS1, mature green stage fruit; FS2, breaker stage fruit; and FS3, red-ripe stage fruit. Different letters indicate significantly different values (*p* < 0.01), as determined by two-way ANOVA followed by Duncan’s multiple range tests.

Anthocyanin contents were quantified in the leaves, stems, flowers and fruits at each stage in these two pepper cultivars (Figure 2). The anthocyanin contents were essentially consistent with the visible pigmentation patterns: anthocyanin levels were high in the stems, flowers and FS2 stage fruits of the P cultivar. These results suggest that the anthocyanin contents of stems, flowers and fruits are responsible for the differences in the purple coloration of the pepper cultivars.

### 2.2. CaAN2 and CaTT8 Are Highly Expressed in Purple Chili Pepper Fruit

**Figure 3:**
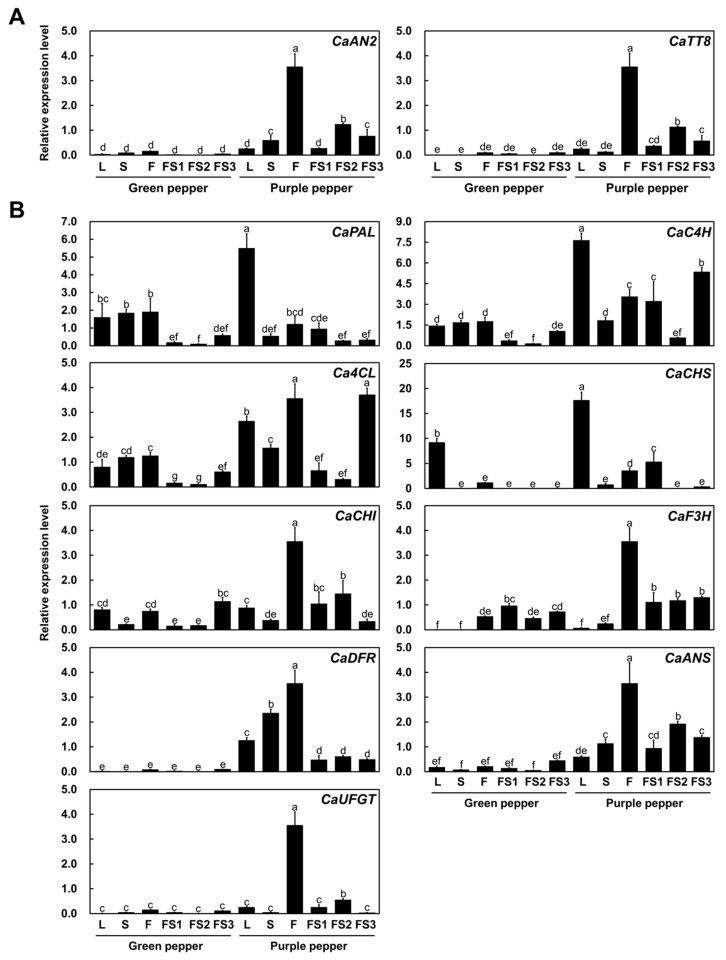
Relative transcript levels of *CaAN2* and other anthocyanin biosynthetic genes from green and purple peppers. Expression levels of anthocyanin regulatory genes (**A**) and anthocyanin biosynthetic genes (**B**). Results represent means ± SD from three independent biological replicates. *CaACTIN* was used as the reference gene. Different letters indicate significantly different values (*p* < 0.01), as determined by two-way ANOVA followed by Duncan’s multiple range tests.

To examine the expression of genes encoding two regulators of anthocyanin biosynthesis, the R2R3 MYB transcription factor gene *CaAN2* and the basic helix-loop-helix (bHLH) transcription factor gene *TRANSPARENT TESTA8* (*CaTT8*), quantitative reverse-transcription PCR (qRT-PCR) was performed with various tissues including leaves, stems, flowers and fruits at different developmental stages in both cultivars. *CaAN2* and *CaTT8* were expressed at higher levels in all tissues of the P vs. G cultivar and their expression levels reflected the extent of pigment accumulation in these tissues (Figure 3A). Specifically, *CaAN2* and *CaTT8* transcript levels were highest in flowers and purple fruit at breaker stage (FS2) in the P cultivar. Indeed, anthocyanin content was correlated with the simultaneous expression of *CaAN2* and *CaTT8*. These results suggest that *CaAN2* and CaTT8 cooperatively regulate anthocyanin biosynthesis in various tissues of chili pepper.

As shown in Figure 3B, the general phenylpropanoid biosynthetic genes *phenylalanine ammonia-lyase* (*CaPAL*), *cinnamate 4*-*hydroxylase* (*CaC4H*) and *4*-*coumarate coenzyme A:ligase* (*Ca4CL*) were highly expressed in the leaves of the P cultivar. However, early biosynthetic genes, including *chalcone synthase* (*CaCHS*), *chalcone* *isomerase* (*CaCHI*) and *flavanone hydroxylase* (*CaF3H*), were highly expressed in flowers of the P cultivar. The late biosynthetic genes, including *dihydroflavonol 4*-*reductase* (*CaDFR*), *anthocyanidin synthase* (*CaANS*) and *UDP*-*flavonoid glucosyl transferase* (*CaUFGT*), were highly upregulated in flowers and fruits (at stage FS2) of the P cultivar, which showed high anthocyanin contents. Taken together, these results confirm that anthocyanin accumulation reflects the expression levels of flavonoid biosynthetic genes across various tissues of different cultivars and that this expression occurs in tissues co-expressing *CaAN2* and *CaTT8*.

### 2.3. Isolation of CaAN2 cDNA and Phylogenetic Analysis

To investigate the mechanism regulating anthocyanin biosynthesis in purple chili pepper, the R2R3-type transcription factor gene *CaAN2* was cloned from P cultivar leaves by PCR. The cDNA sequence of *CaAN2* was 100% identical to the previously reported sequence for *CaAN2*, comprising a 789-bp coding sequence encoding a predicted protein of 262 amino acids (GenBank accession number NP_001311547.1)

**Figure 4:**
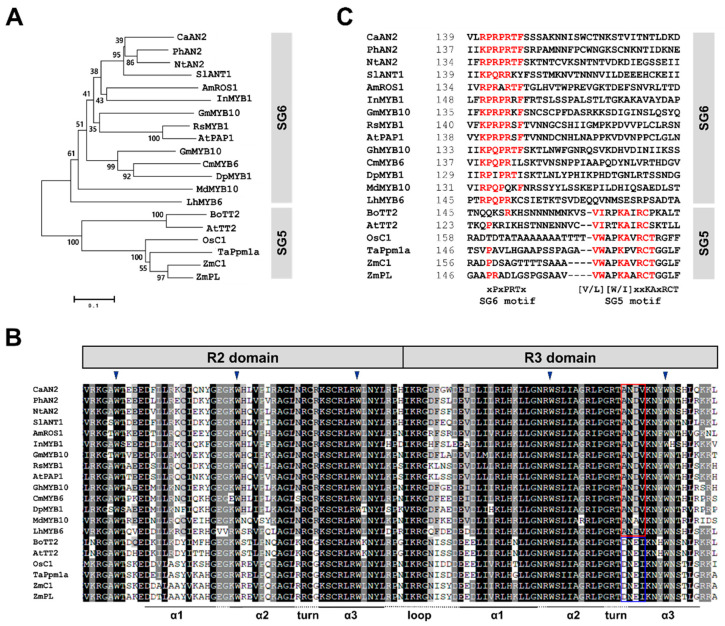
Phylogenetic analysis and multiple sequence alignment of anthocyanin-activating R2R3 MYBs. (**A**) Phylogenetic tree of pepper *CaAN2* and R2R3 MYB proteins from other plants. The phylogenetic tree was constructed using the neighbor-joining method with MEGA6 software. The GenBank accession numbers of species used in this study are listed in Supplementary Table S1. (**B**) Multiple sequence alignment of the R2 and R3 domains across the R2R3 MYB proteins shown in (**A**). The conserved residues ANDV and DNEI are represented by red and blue boxes, respectively. Inverted blue triangles indicate the conserved residues forming the inner hydrophobic core of the R2 and R3 domains. (**C**) Multiple sequence alignment of parts of the C-terminal regions of R2R3-MYB sequences, showing the SG5 and SG6 motifs. Amino acids matching either the SG6 or SG5 motif are indicated in red. The starting amino acid position of each sequence is given in the second column.

Flavonoid-related MYB transcription factors belonging to SG5 and SG6 regulate anthocyanin biosynthesis in different tissues of various plants, including leaves, fruits and seeds [11,15,16]. In the phylogenetic tree generated from R2R3-MYB proteins from various plant species (Figure 4A), *CaAN2* falls into the SG6 clade, together with PhAN2, AtPAP1 and MdMYB10: these eudicot MYB transcription factors actively regulate anthocyanin biosynthesis [16,17,18,19]. Sequence alignments showed that all SG5 and SG6 R2R3-MYBs share the highly conserved motif [D/E]Lx2[R/K]x3Lx6Lx3R in the R3 domain, which is functionally important for interactions between MYB and R/B-like bHLH proteins [11,16,19]. Additionally, the R2R3 domain of *CaAN2* contains five conserved tryptophans that are important for forming the helix-loop-helix protein architecture at the N terminus. At the end of the R3 domain, *CaAN2* harbors the conserved ANDV motif, a characteristic feature of SG6 R2R3-MYBs (Figure 4B). While *CaAN2* contains this conserved SG6 motif ([K/R]P[Q/R]P[Q/R]TF), it also harbors a highly variable C-terminal region compared to those of other anthocyanin-activating R2R3 MYB transcription factors [11,15,17] (Figure 4C). These results suggest that *CaAN2* activates anthocyanin biosynthesis in chili pepper.

### 2.4. CaAN2 Is an Active Regulator of Anthocyanin Biosynthesis

**Figure 5:**
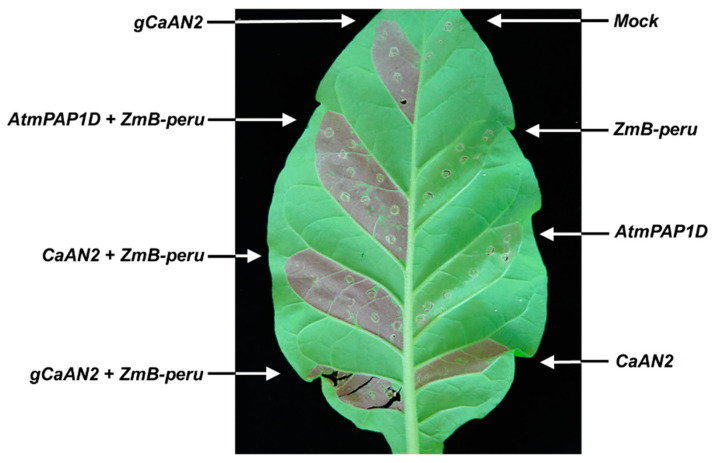
Anthocyanin accumulation in a tobacco leaf transiently infiltrated with *Agrobacterium* expressing *CaAN2* and other anthocyanin biosynthesis regulatory genes. Tobacco leaves were transiently infiltrated with *Agrobacterium* cultures carrying empty vector or the indicated combinations of constructs harboring *ZmB-peru*, *AtmPAP1* and *CaAN2*. A representative photograph of a transiently infiltrated tobacco leaf at 5 days after agroinfiltration is shown.

To evaluate the role of *CaAN2* in anthocyanin biosynthesis, various combinations of diverse transcription factor genes, including genes encoding a bHLH *B-peru* from (*ZmB-peru*), R2R3 MYB *mPAP1D* from Arabidopsis (*AtmPAP1D*) and *CaAN2*, were transiently expressed in tobacco leaves (Figure 5). Individual infiltration with the *ZmB-peru AtmPAP1D* gene did not induce anthocyanin production, whereas co-infiltration with both genes induced anthocyanin accumulation in tobacco leaves. By contrast, the transient overexpression of the full-length genomic sequence or cDNA of *CaAN2* induced visible pigment accumulation in the absence of the bHLH transcription factor gene *ZmB-Peru*, as did the simultaneous expression of *CaAN2* and *ZmB-peru*. These results indicate that *CaAN2* is a positive regulator of anthocyanin biosynthesis.

### 2.5. CaAN2 Is a Good Candidate Visible Selectable Marker Gene

**Figure 6:**
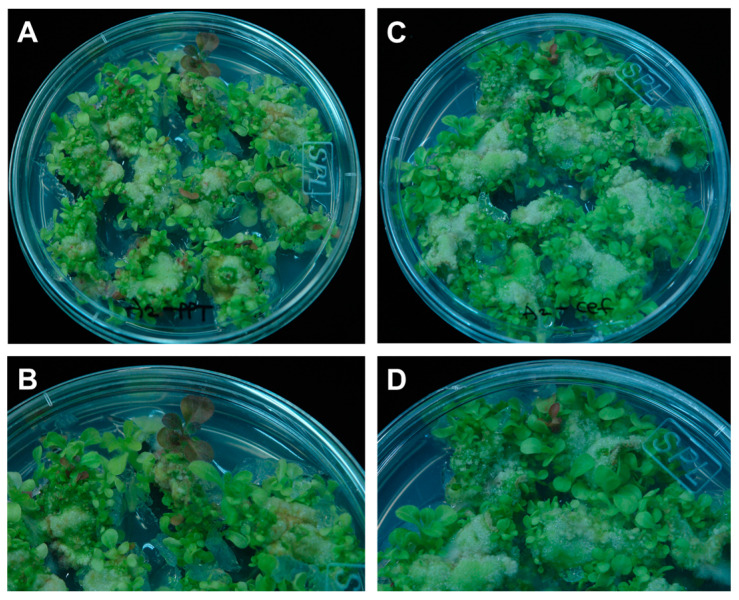
*CaAN2* expression in tobacco leaf explants enables the easy visual screening of transgenic tobacco plants. Purple coloration caused by anthocyanin accumulation can be observed in leaves grown on medium with (**A**,**B**) or without PPT (**C**,**D**).

As shown in the transient assay, pigment accumulation was readily detected by the expression of *CaAN2*. To verify the utility of this gene as a visible marker, we transformed tobacco leaf disks with *Agrobacterium* containing *pB2GW7*-*CaAN2*, which includes the *bar* gene for phosphinothricin (PPT) resistance and cultured the resulting explants with or without PPT. Green shoots were detected more on PPT-free medium than on medium containing PPT (Figure 6). Additionally, purple shoots were observed on both PPT and PPT-free medium. Regenerated plantlets harboring the *CaAN2* gene showed distinct red coloration and were easily detected from the callus phase to plant regeneration. These results suggest that pigment accumulation via the expression of an anthocyanin activating regulator can be used to select transgenic plants without the need for selective pressure from antibiotics or herbicides.

### 2.6. Ectopic Expression of CaAN2 Strongly Promotes Anthocyanin Biosynthesis

**Figure 7:**
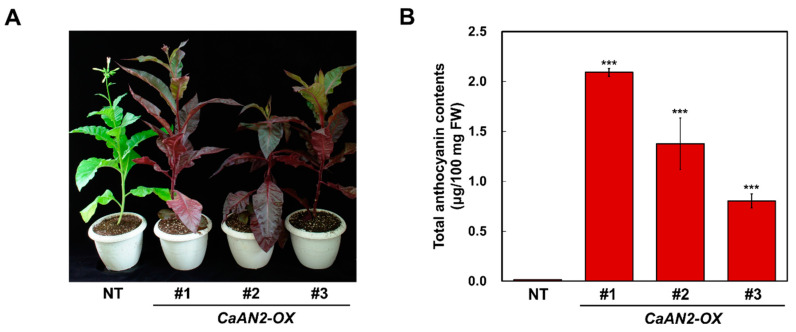
Phenotypes and relative anthocyanin contents of transgenic tobacco plants containing *CaAN2*. (**A**) Phenotypes of transgenic plants. (**B**) Total anthocyanin contents. NT, nontransgenic tobacco plant; *CaAN2-OX*, transgenic tobacco plants ectopically expressing *CaAN2*. All results represent mean values ± SD from three independent biological replicates. Asterisks indicate values that differ significantly from NT at *p* < 0.001 according to a Student’s paired *t*-test.

Transgenic tobacco plants harboring *CaAN2* showed easily distinguishable pigmentation throughout the plant body, including leaves, stems and flowers (Figure 7A). The transgenic tobacco plants showed high anthocyanin contents, whereas the non-transgenic (NT) tobacco plants did not, indicating that *CaAN2* strongly increased anthocyanin production and conferred an intense red-purple color due to strong anthocyanin accumulation (Figure 7B). Taken together, these results demonstrate that heterologous expression of *CaAN2* strongly enhances anthocyanin accumulation and confers an intense red-purple color in tobacco.

**Figure 8:**
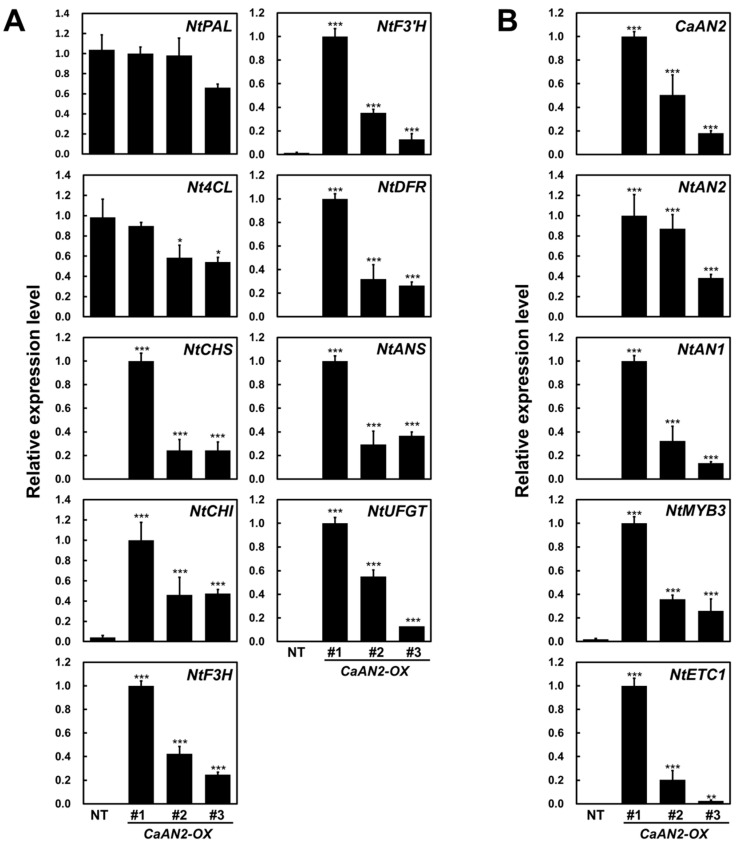
Expression profiles of anthocyanin biosynthetic genes and endogenous transcription factor genes in the leaves of nontransgenic tobacco (NT) and three independent transgenic tobacco lines harboring *CaAN2*. The relative transcript levels of anthocyanin biosynthetic genes (**A**) and regulatory genes (**B**) were measured by qRT-PCR, with *NtGAPDH* used as a reference gene. The biosynthetic pathway genes evaluated include those encoding *chalcone synthase* (*NtCHS*), *chalcone isomerase* (*NtCHI*), *flavanone 3-hydroxylase* (*NtF3H*), *flavonoid 3′-hydroxylase* (*NtF3′H*), *dihydroflavonol 4-reductase* (*NtDFR*), *anthocyanidin synthase* (*NtANS*) and *UDP-flavonoid glucosyltransferase* (*NtUFGT*) as well as the upstream enzyme *phenylalanine ammonia-lyase* (*NtPAL*) and *4-coumarate-CoA ligase* (*Nt4CL*). Results are means ± SD from three independent biological replicates. *, ** and *** indicate values that differ significantly from NT at *p* < 0.05, *p* < 0.01 and *p* < 0.001, respectively, according to a Student’s paired *t*-test.

qRT-PCR was performed to investigate the expression of anthocyanin biosynthetic genes and related transcriptional regulators in transgenic tobacco plants harboring *CaAN2*. To examine the relationship between leaf color and the transcript levels of anthocyanin biosynthetic pathway genes, the expression of nine structural genes, including the upstream genes *NtPAL* and *Nt4CL*; the early biosynthetic genes *NtCHS*, *NtCHI*, *NtF3H* and *flavonoid 3′*-*hydroxylase* (*NtF3′H*); and the late biosynthetic genes *NtDFR*, *NtANS* and *NtUFGT* was measured. The expression of anthocyanin biosynthesis regulatory genes, including the R2R3-MYB activator gene *NtAN2*, the bHLH activator gene *NtAN1*, the R2R3-MYB repressor gene *NtMYB3* and the R3 repressor gene *NtETC1* was also analyzed. The expression levels of all structural genes except *NtPAL* and *Nt4CL* were higher in transgenic vs. NT plants (Figure 8A). The transcript levels of these upregulated genes were similar to those of *CaAN2*. As expected, *CaAN2* transcripts were only present in transgenic plants but not in NT plants. In addition, the transcript levels of *NtAN1* and *NtAN2*, encoding endogenous anthocyanin biosynthesis activators, were high in transgenic plants but not in NT plants. Additionally, the transcript levels of the R2R3-MYB type repressor *NtMYB3* and the R3-MYB type repressor *NtETC1* were high in transgenic tobacco plants but not in NT plants (Figure 8B). These results indicate that the ectopic expression of *CaAN2* promotes the transcription of anthocyanin-biosynthesis-related regulators and biosynthetic genes, resulting in anthocyanin accumulation in tobacco leaves.

### 2.7. Plant Coloration Facilitates Detection of and Selection against Chimerism

Chimerism is a fairly common occurrence during plant transformation and is a prime factor in the failure to transfer genes to subsequent generations [20]. Therefore, minimizing chimerism is indispensable for establishing an efficient and reliable transformation system. Here, transformed shoots with overall uniform red coloration were selected and progressed these shoots to subsequent generations. After the next several generations, *CaAN2* was stably transmitted, resulting in strong pigmentation due to anthocyanin accumulation. This process will be useful for reducing the occurrence of chimerism during transformation using *CaAN2* as a visible marker gene.

## 3. Discussion

The molecular genetic improvement of crops is strongly dependent on the selection of transformants with the desired traits. Selectable markers for antibiotic or herbicide resistance are commonly used for the screening of transformants. However, public concerns have been raised about the presence of selectable markers due to possible risks for human health and the environment [3]. Therefore, several alternative methods have been developed to generate marker-free transgenic plants, such as co-transformation and segregation, site-specific recombinase-mediated excision and intrachromosomal homologous recombination, but these techniques are costly, time-consuming and/or inefficient [21]. Additionally, plant selection systems consist of two components: chemical agents and selectable marker genes. During the tissue culture process, chemicals such as herbicides or antibiotics must be added to the tissue culture medium.

Anthocyanins, a group of flavonoid metabolites derived from phenylpropanoid compounds, are widely present in various plant tissues, including leaves, stems, flowers and fruits [7]. Additionally, high anthocyanin content in foods is generally considered beneficial to human health due to their strong antioxidant properties. Tissues containing anthocyanins can easily be discerned with the naked eye without the need for additional treatments. To utilize anthocyanin as a visible marker, it is essential to identify key genes for anthocyanin biosynthesis. In this study, the role of *CaAN2* from purple fruited chili pepper in anthocyanin pigment accumulation was verified. The high expression of *CaAN2* activated the transcription of anthocyanin biosynthetic genes from pepper as well as tobacco, resulting in pigment accumulation (Figure 2 and Figure 5). Some anthocyanin-activating R2R3 regulators indispensably require bHLH transcription factors to induce anthocyanin accumulation [22]. Mangosteen (*Garcinia mangostana*) GmMYB10 did not activate the *AtAFR* or *GmDFR* promoter when expressed alone in a transient expression assay, but it activated these genes when co-expressed with AtbHLH2 [23]. In addition, transgenic Lisianthus containing snapdragon *AmROSEA1* (R2R3 MYB) showed anthocyanin accumulation only in sepals, which strongly express a bHLH cofactor gene [24]. Transgenic tobacco plants simultaneously expressing the maize *ZmB-peru* gene and the Arabidopsis *AtmPAP1D* gene displayed notable color changes compared to plants individually expressing *ZmB-peru* or *AtmPAP1D* [22]. Here, we determined that *CaAN2* alone is sufficient to activate anthocyanin biosynthesis in tobacco and pepper; therefore, it can be used as a visible reporter gene for plant transformation. Evaluation of the effect of *CaAN2* in a heterologous tobacco system confirmed that this gene can be useful for identifying transgenic plants without the need for the expensive equipment required for GFP detection or the chemical staining required for GUS detection.

The use of the genome-editing tool CRISPR-Cas9 for the molecular breeding of crops is on the rise. In addition, molecular characterization of transgene-free gene-edited plants is required and this is a time-consuming and labor-intensive process. The *OsC1* reporter gene is a valuable tool to aid in the visible screening of transformants at high efficiency [25]. The combination of Cas9 protein and *OsC1* represents a powerful selection system for transgenic rice plants, allowing transgene-free, gene-edited plants to be easily selected on the basis of a color change. Advanced methods based on anthocyanin accumulation can enable the robust, rapid selection of plants with gene-edited target traits.

Here, we successfully used *CaAN2* to monitor the transient transformation of tobacco and successfully selected transformed cells. The selection of transformed plants is generally labor- and time-intensive [26]. However, the expression of *CaAN2* can easily be monitored in transgenic plants based on anthocyanin accumulation. The application of *CaAN2* for CRISPR-Cas9-mediated genome editing is feasible for the selection of transgenic or transgenic-free genome-edited plants. Using this method, transgenic plants could be screened without the need for additional treatments.

## 4. Materials and Methods

### 4.1. Plant Materials

Two pepper (*Capsicum annuum*) cultivars, the green cultivar ‘AG188’ and the purple cultivar ‘20GP15-2’, were used in this study; these cultivars are referred to as G and P, respectively. The seeds were obtained from the National Institute of Horticultural and Herbal Science (Wanju, Korea) and cultivated in the greenhouse. Tobacco (*Nicotiana tabacum* cv. Xanthi) plants were grown in greenhouses and growth chambers at Hankyong University (Anseong, Korea) under natural light at 26  ±  2 °C and used for transient *Agrobacterium* (*Agrobacterium tumefaciens*)-mediated infiltration assays and stable transformation.

To analyze anthocyanin contents and the transcript levels of anthocyanin biosynthetic genes, all samples were rapidly frozen in liquid nitrogen and stored at −80 °C. Each sample was ground to a powder and split into two aliquots: one for RNA extraction and the other to measure anthocyanin contents.

### 4.2. RNA Extraction, cDNA Synthesis and Isolation of Genomic DNA

Total RNA was extracted from various tissues of both pepper cultivars and from tobacco leaves using TRIzol reagent (Invitrogen, Carlsbad, CA, USA) and purified using a FavorPrep Plant Total RNA Mini Kit (Favorgen, Changzhi, Taiwan) according to the manufacturer’s instructions. First-strand cDNA was synthesized from 2 μg total RNA using amfiRivert cDNA Synthesis Platinum Master Mix (GenDEPOT, Barker, TX, USA) for qRT-PCR analysis. Genomic DNA was extracted from the samples with a DNeasy Plant Mini Kit (Qiagen, Valencia, CA, USA) according to the manufacturer’s instructions.

### 4.3. Measurement of Total Anthocyanin Contents

Total anthocyanin contents in frozen tissue samples were measured as previously described [27]. Each 100 mg (fresh weight) tissue sample was incubated in 600 μL extraction buffer (methanol containing 1% [*v/v*] HCl) for 6 h at 4 °C with moderate agitation. After the addition of 200 μL water and 200 μL chloroform, the sample was centrifuged at 14,000× *g* for 5 min at 4 °C to precipitate the plant debris. The absorbance of the supernatant was recorded at 530 nm (A_530_) and 657 nm (A_657_) using a microplate reader. Anthocyanin contents were determined according to the formula A_530_ − (0.25 × A_657_). Each sample was extracted and examined using three independent experiments.

### 4.4. Quantitative Reverse-Transcription PCR (qRT-PCR) Analysis

Transcript levels were measured by qRT-PCR using AccuPower 2x Greenstar qPCR Master Mix (Bioneer, Daejun, Korea) and a Bio-Rad CFX96 Detection System (Bio-Rad Laboratories, Hercules, CA, USA) according to the manufacturer’s instructions. Gene expression levels were normalized to *actin* (*CaACTIN*) and *glyceraldehyde 3-phosphate dehydrogenase* (*NtGAPDH*) for pepper and tobacco, respectively, as the reference gene. Three independent biological replicates were performed per sample. The primers used for RT-qPCR analysis are listed in Supplementary Table S2.

### 4.5. Gene Isolation and Sequence Analysis

The full-length coding sequence of *CaAN2* was amplified from cDNA and genomic DNA from purple pepper by PCR with PrimeSTAR HS DNA Polymerase (Takara, Otsu, Japan) using the primer pair *CaAN2* F/R (Supplementary Table S1). All amplicons were subcloned into the pENTR/D-TOPO vector (Invitrogen) for validation by sequencing. Multiple sequence alignments were generated using the CLUSTALW program (https://www.genome.jp/tools-bin/clustalw). A phylogenetic tree was constructed using the neighbor-joining method [28] with MEGA version 6 software [29].

### 4.6. In Planta Assays of the Anthocyanin Biosynthesis Activity of CaAN2

Amplified DNA products of *CaAN2* were cloned into the Gateway entry vector pDONR221 (Invitrogen) using PCR-specific primer sets (Supplementary Table S1) and incorporated into the Gateway destination vector pB2GW7 (VIB-Ghent University, Ghent, Belgium) via several Gateway cloning steps. The resulting constructs were introduced into *Agrobacterium* strain GV3101 for the transient infiltration assay and stable transformation.

To perform the tobacco agroinfiltration assay, *Agrobacterium* cultures harboring pB2GW7-*CaAN2*, pB2GW7-*ZmB-peru* and pB2GW7-*AtmPAP1* were grown in LB medium at 28 °C with shaking until the optical density at 600 nm reached 1.2. The bacteria were pelleted and resuspended in infiltration solution containing 10 mM MgCl_2_ and 0.1 mM acetosyringone. Equal amounts of *Agrobacterium* suspensions harboring each construct were infiltrated into the abaxial surfaces of the expanded leaves of 6-week-old tobacco plants as described [28]. Photographs of infiltrated leaves were taken at 5 days after infiltration.

### 4.7. Plant Regeneration

Transgenic tobacco (*N. tabacum* cv. Xanthi) plants were generated by transformation with *Agrobacterium* containing the pB2GW7-*CaAN2* construct using the leaf disc method [22]. Briefly, tobacco seeds were surface sterilized and grown on solidified half-strength Murashige and Skoog (MS) medium (Duchefa, Haarlem, Netherlands). The plants were grown in a growth chamber under a 16 h light/8 h dark cycle at 26 ± 1 °C for 2 months. Leaf discs were obtained from the cultured plants and submerged in *Agrobacterium* mixture. To identify transgenic events, explants were cultured on shoot-inducing medium with or without 10 mg/L phosphinothricin (PPT, Duchefa). The regenerated shoots were subsequently transferred to MS medium to enable rooting and cultivated in a greenhouse to maturity. Three representative tobacco lines were selected for further analysis. Transgenic T_3_ lines were developed by successive self-pollination of T_0_ plants.

## 5. Conclusions

In this study, we characterized *CaAN2* as a common key regulator of anthocyanin pigmentation in purple chili pepper, cooperatively expressed with *CaTT8*. Through the transient assay and stable tobacco transformation, it confirmed that individual expression of *CaAN2* sufficiently induced the anthocyanin accumulation in a heterologous system. Additionally, anthocyanin accumulated phenotypes were stably inherited into the next generation without chimerism. Taken together, it indicates that *CaAN2* is useful as an alternative selection system for transgenic plants, as a visible selective marker gene.

## Data Availability

The data presented in this study, are available on request from the corresponding authors. The data are not publicly available because their elaboration are all reported in the manuscript.

## Supplementary Materials

The following supporting information can be downloaded at: https://www.mdpi.com/article/10.3390/plants11060820/s1, Table S1: List of primers used in this study. Table S2: List of primers used in this study.
