# Supplementary material for: Chili Pepper AN2 (CaAN2): A Visible Selection Marker for Nondestructive Monitoring of Transgenic Plants
Source: Plants (Basel). 2022 Mar 19;11(6):820. doi: 10.3390/plants11060820 (PMC8955877; doi:10.3390/plants11060820)
Supplement: Supplementary file 1 [file plants-11-00820-s001.zip › Supplementary Table (CaAN2)_modified.pdf]

Supplementary Table S1. Anthocyanin-related R2R3-MYB regulators from various species

R2R3 regulator	Species	Accession number
AmROS1	*Antirrhinum majus*	ABB83826
AtPAP1	*Arabidopsis thaliana*	AAG42001
AtTT2	*Arabidopsis thaliana*	CAC40021
BoTT2	*Brassica oleracea*	ADP76653.1
CmMYB6	*Chrysanthemum morifolium*	KR002097.1
DpMYB1	*Dahlia pinnata*	AB601003
GmMYB10	*Garcinia mangostana*	ACM62751.1
GhMYB10	*Gerbera hybrida*	CAD87010
InMYB1	*Ipomoea nil*	BAE94391
LhMYB6	*Lilium hybrida*	BAJ05399
MdMYB10	*Malus × domestica*	ACQ45201
NtAN2	*Nicotiana tabacum*	ACO52470.1
OsC1	*Oryza sativa*	BAD04024
PhAN2	*Petunia × hybrida*	AAF66727
RsMYB1	*Raphanus sativus*	AKM95888.1
SlANT1	*Solanum lycopersicum*	AAQ55181.1
TaPpm1	*Triticum aestivum*	MG066451
ZmC1	*Zea mays*	AAA33482
ZmPL	*Zea mays*	AAA19819

Supplementary Table S2. List of primers used in this study.

Usage	Primer name	Primer sequence (5'-3')
Gene expression	qRT-CaAN2-F	AGATTGCCGGGAAGAACAGCAAAC
	qRT-CaAN2-R	TTGCACTTGATGAGAAGGTCCGAG
	qRT-CaTT8-F	GCCAAAATTCAGACAGTGGT
	qRT-CaTT8-R	TCTTCTTCGTGCTCTTCGTT
	qRT-CaPAL-F	ATTGATTTTTGCAAGAAATCAATTC
	qRT-CaPAL-R	GCTCCACTTTAGCCCCAC
	qRT-CaC4H-F	GATTCCTTCCATTCGGTGTT
	qRT-CaC4H-R	CCTTTCTCCGTGGTGTCG
	qRT-Ca4CL-F	CTGGACCAGTGCTGGCAAT
	qRT-Ca4CL-R	GGTTACGGGGCAAAGAACAA
	qRT-CaCHS-F	GTGGAACCGTTATCCGACTAGCAA
	qRT-CaCHS-R	GTATCACTTGGGCCACGGAAAGTA
	qRT-CaCHI-F	CCTCCTGGTTCTACACCACC
	qRT-CaCHI-R	CTTTGCGGCAGGTGAAACTC
	qRT-CaF3H-F	ACGCTGATCATCAAGCAGTG
	qRT-CaF3H-R	CTGAAGAGGTTGCCGAAAAG
	qRT-CaDFR-F	CAAGGCAGAGGGAAGATTCA
	qRT-CaDFR-R	TCTGTCGGCAAGTCTCAATG
	qRT-CaANS-F	CAAATGCCCACAACCAGAACTAGC
	qRT-CaANS-R	CGCACTTTGCAGTTACCCACTTTC
	qRT-CaUFGT-F	CCTGAGAGTCATGTACATGGAGG
	qRT-CaUFGT-R	TCCAACTAATTCCTAGTTCCTCGG
	qRT-CaACTIN-F	CCACCTCTTCACTCTCTGCTCT
	qRT-CaACTIN-R	ACTAGGAAAAACAGCCCTTGGT
	qRT-NtPAL-F	ATTGAGGTCATCCGTTCTGC
	qRT-NtPAL-R	ACCGTGTAACGCCTTGTTTC
	qRT-Nt4CL-F	TCATTGACGAGGATGACGAG
	qRT-Nt4CL-R	TGGGATGGTTGAGAAGAAGG

	qRT-NtCHS-F	TTGTTCGAGCTTGTCTCTGC
	qRT-NtCHS-R	AGCCCAGGAACATCTTTGAG
	qRT-NtCHI-F	GTCAGGCCATTGAAAAGCTC
	qRT-NtCHI-R	CTAATCGTCAATGCCCCAAC
	qRT-NtF3H-F	CAAGGCATGTGTGGATATGG
	qRT-NtF3H-R	TGTGTCGTTTCAGTCCAAGG
	qRT-NtF3'H-F	AGGCTCAACACTTCTCGT
	qRT-NtF3'H-R	CATCAACTTTGGGCTTCT
	qRT-NtDFR-F	AACCAACAGTCAGGGGAATG
	qRT-NtDFR-R	TTGGACATCGACAGTTCCAG
	qRT-NtANS-F	TGGCGTTGAAGCTCATACTG
	qRT-NtANS-R	GGAATTAGGCACACACTTTGC
	qRT-NtUFGT-F	CAATGTTTGGGATGGTGTCA
	qRT-NtUFGT-R	TTCCTCCTCTGCCTCTTTCA
	qRT-NtAN2-F	GTAGACTTCCTGGAAGGACGGCAA
	qRT-NtAN2-R	GGCCGAGGTCTGAATATGGTGATC
	qRT-NtAN1-F	CTTGAACACTTCTCAAACCGA
	qRT-NtAN1-R	TGCTAGGGCACAATGTGAAG
	qRT-NtMYB3-F	CCGGGGAGAACTGATAATGA
	qRT-NtMYB3-R	TTTCTGGCCAAAACTCAAGG
	qRT-NtETC1-F	TCCTCCTGATTCTCAAGGAAA
	qRT-NtETC1-R	TCCGGGTATTCTTCCAGCTA
	qRT-NtGAPDH-F	GGTGTCCACAGACTTCGTGG
	qRT-NtGAPDH-R	GACTCCTCACAGCAGCACCA
Gene cloning	CaAN2-F	ATGAATACTGCTATTATTGCCAAGTCCTCTG
	CaAN2-R	CTAATTAAGTAGATTCCATAGGCCAATATCAG
	pDONR-CaAN2-F	AAAAAAGCAGGCTCCATGAATACTGCTATTATTG
	pDONR-CaAN2-R	GTACAAGAAAGCTGGGTCCTAATTAAGTAGATTC
